# What are the Training Needs of Mental Health and other Healthcare Professionals in Women’s Mental Health?

**DOI:** 10.1192/j.eurpsy.2022.166

**Published:** 2022-09-01

**Authors:** E. Sönmez Güngör

**Affiliations:** Erenköy Mental Health Training and Research Hospital, Psychiatry, İstanbul, Turkey

**Keywords:** women’s mental health, training in psychiatry, perinatal mental health

## Abstract

Women’s mental health deals with, but not limited to, various aspects of psychiatric disorders in women, across the lifespan. The differential influence of gender and gender related indirect factors on mental ill-health has been researched and established for at least three decades. However, this topic is seldomly considered to be included in the structured curricula of psychiatric training. Several institutions provide temporary, CME-linked courses and other training opportunities. Yet, in many countries it is not part of a core program, whereas in common mental disorders, it is women who predominate. In this talk, a special focus will put on the training needs of psychiatrists and other mental health professionals regarding women’s mental health, especially perinatal mental health and psychopharmacology during pregnancy and lactation. Another important aspect is the impact of intimate partner violence on the mental (and general) wellbeing of women, which despite being encountered by around 1 out of 3 women worldwide, is still poorly addressed and treated. Some other contemporary aspects such as the mental health of immigrant women, will also be discussed.

**Disclosure:**

No significant relationships.

